# Decontamination from water pollutants and pathogens by electrospun nanofibers doped with heavy-atom-free borafluorene-BODIPY photosensitizers

**DOI:** 10.3762/bjnano.17.46

**Published:** 2026-05-20

**Authors:** Angelika Zaszczyńska, Paulina H Marek-Urban, Karolina Wrochna, Agnieszka E Kuklewska, Kacper Kręgielewski, Marta Grodzik, Dawid R Natkowski, Jolanta Mierzejewska, Ewa Iwanek, Agata Blacha-Grzechnik, Paweł Sajkiewicz, Krzysztof Durka

**Affiliations:** 1 Institute of Fundamental Technological Research, Polish Academy of Sciences, Pawińskiego 5b St., 02-106 Warsaw, Poland; 2 Faculty of Chemistry, Warsaw University of Technology, Noakowskiego 3, 00-664 Warsaw, Poland; 3 Department of Nanobiotechnology, Institute of Biology, Warsaw University of Life Sciences (WULS-SGGW), 02-787 Warsaw, Poland; 4 Faculty of Chemistry, Silesian University of Technology, Strzody 9, 44-100 Gliwice, Poland; 5 Centre for Organic and Nanohybrid Electronics, Silesian University of Technology, Konarskiego 22B, 44-100 Gliwice, Poland

**Keywords:** antimicrobial photodynamic therapy, BODIPY, electrospun nanofibers, heavy-atom free photosensitizers, immobilization, polycaprolactone, singlet oxygen, water purification

## Abstract

A heavy-atom-free and non-toxic spirocyclic C-BODIPY singlet oxygen photosensitizer was successfully incorporated into electrospun polymeric nanofibers. Optimization of the material composition revealed that polycaprolactone (PCL), an FDA- and EMA-approved, biodegradable, easily accessible, and cost-efficient polymer, doped with BODIPY at a concentration of only 0.15 wt %, is an efficient photocatalyst for the degradation of the pharmaceutical agents ranitidine, propranolol, and cimetidine, selected as model water pollutants. The obtained nanofibers showed smooth and uniform morphology along with very high durability and resistance toward oxidation, remaining active even after 20 reaction cycles. EDX, ToF-SIMS and XPS analyses confirmed the homogenous distribution of BODIPY within the polymeric matrix. Furthermore, the materials showed significant photoinactivation of *Staphylococcus aureus* under white light irradiation compared to the control experiment performed without irradiation. These findings highlight the potential of the electrospun PCL nanofibers as optimal matrix for the immobilization with singlet oxygen photosensitizers and subsequent application in the decontamination of water from pollutants and pathogens.

## Introduction

Due to the enormous population, economic, and industrial growth, the rapid rise in demand for water resources has become one of the major contemporary issues that needs to be addressed in the following decades [1]. A huge increase of organic compound production by chemical, textile, food, and pharmaceutical industries results in harming aquatic ecosystems and even further reduces the available freshwater resources. Relatedly, bacteria, fungi, and viruses continuously exposed to various cytotoxic biologically active compounds flushed to water bodies from the pharmaceutical industry and farms developed resistance mechanisms, becoming more harmful to humans, animals, and cultivations. One promising method for wastewater treatment employs photochemical processes that utilize photosensitizers, that is, light-activated molecules producing reactive oxygen species (ROS), such as singlet oxygen (^1^O_2_), capable of degrading organic pollutants or microbes [2–6].

**Figure 1:**
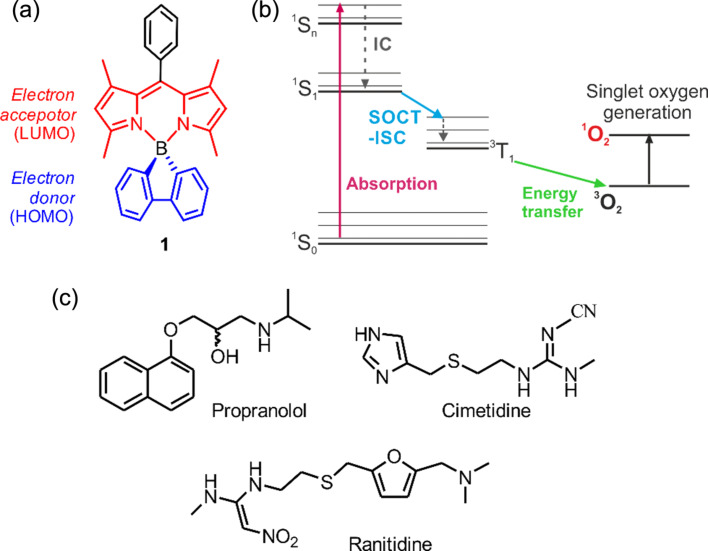
(a) Structure of studied BODIPY photosensitizer **1**. (b) Energy diagram presenting the light-induced production of singlet oxygen by a triplet photosensitizer. (c) Chemical structures of the target pollutants.

Among various groups of organic photosensitizers, boron dipyrromethenes (BODIPYs, i.e., 4,4-diﬂuoro-4-bora-3a,4a-diaza-*s*-indacene and its derivatives) [7–13] have attracted considerable interest due to their advantageous properties, including strong light absorption, facile synthesis, and high structural tunability [14–16]. Yet, the main design strategy for photosensitizing BODIPYs is to incorporate heavy atoms (e.g., I, Ru, Pt, and Au) [17–20], which enhance intersystem crossing induced by spin–orbit coupling (SOC-ISC), but also increase dark toxicity, shorten the triplet lifetime, and decrease photostability [21–23]. For these reasons, heavy-atom-free BODIPY photosensitizers have been developed [24–31]. Recently, we proposed spiranic donor–acceptor C-BODIPY derivatives based on 9-borafluorene, bis[1]benzothieno[1,4]thiaborine, or 3,6-diaza-9-borafluorene scaffolds, where the boron atom plays the role of a natural separator between donor (organoboron) and acceptor (dipyrromethene ligand) sites (Figure 1a) [32–35]. Importantly, the proposed systems produce triplet states via an alternative to the SOC-ISC mechanism, that is, through a spin–orbit charge transfer intersystem crossing (SOCT-ISC, Figure 1b) resulting from the electron hopping between perpendicularly oriented donor and acceptor units [36–39].

In the current study, we investigate the borafluorene-based BODIPY **1** (Figure 1a) as a model heavy-atom-free photosensitizer for deactivating water pollutants and pathogens. Our major goal was to develop a suitable polymeric platform and processing method giving materials with large surface areas, good mechanical properties, and high porosity, enabling oxygen diffusion, light penetration, and efficient ROS production. Accordingly, we used electrospinning, a relatively simple and cheap technique with the perspective for large-scale industrialized production of membrane filters [40–49]. We optimized electrospinning conditions and material composition using three different polymers, namely, poly(methyl methacrylate) (PMMA), polycaprolactone (PCL), and polystyrene (PS), doped with various concentrations of **1**. The resulting materials were comprehensively characterized and further tested for their efficacy in degrading the active pharmaceutical ingredients (APIs) cimetidine, ranitidine, and propranolol (Figure 1c) and in inactivating a selected pathogen, namely, the Gram-positive bacterium *Staphylococcus aureus*.

## Experimental

### Materials

BODIPY photocatalyst **1** was already available from our previous studies [34]. The purity of the material was confirmed by a repeated measurement of the ^1^H NMR spectrum (Figure S1, Supporting Information File 1). Cimetidine, ranitidine, and propranolol were purchased from Sigma-Aldrich and were used without further purification. Polycaprolactone (PCL, *M*_w_ = 80.000 g·mol^−1^) and polystyrene (PS, *M*_w_ = 35.000 g·mol^−1^) were purchased from Merck, while poly(methyl methacrylate) (PMMA, *M*_w_ = 100.000 g·mol^−1^) was purchased from Polysciences, Inc. The solvents acetic acid (AA), formic acid (FA), *N*,*N*-dimethylformamide (DMF), and tetrahydrofuran (THF), as well as the compounds tetrabutylammonium bromide (TBAB), 10-anthracenediyl-bis(methylene) dimalonic acid (ABDA), cimetidine (98%), ranitidine (99.1%), and propranolol (99%) were purchased from Merck and Acros and were used as received without additional purification.

### Electrospinning process

**Figure 2:**
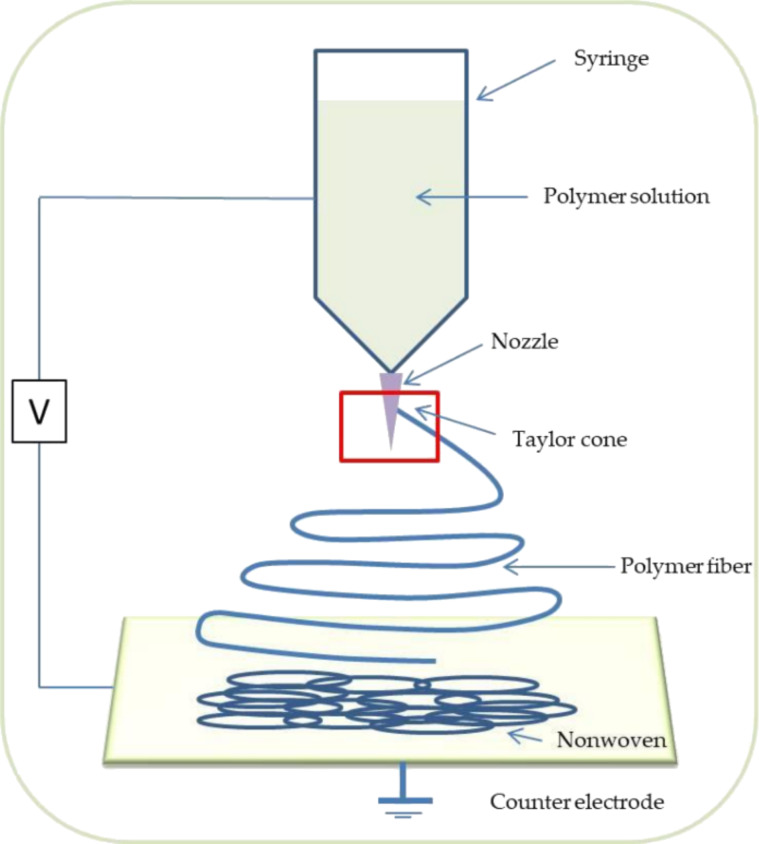
Scheme of the electrospinning process. Figure 2 was reproduced from [50], (© 2021 A. Zaszczynska et al., distributed under the terms of the Creative Commons Attribution 4.0 International License, https://creativecommons.org/licenses/by/4.0).

All samples were electrospun using an electrospinning chamber (FLUIDNATEK® LE-5, Bioinicia, Spain) with air conditioning (Bioinicia, Spain) at room temperature and a humidity of ~35% (Figure 2).

PCL was dissolved in acetic acid and formic acid (1:9, AA/FA), at a concentration of 12 wt %. A high voltage (12 kV) was applied between needle and collector. The distance between the needle and collector was fixed at 100 mm with the feed rate of 1 mL·h^−1^. PMMA was dissolved in DMF at a concentration of 30%. The process parameters, such as voltage (10 kV), tip-to-collector distance (120 mm), and the feed rate (1 mL·h^−1^) were fixed. PS was dissolved in DMF/THF (1:1 ratio) at a concentration of 15%. The process parameters such as voltage (20 kV), tip-to-collector distance (150 mm), and the feed rate (2 mL·h^−1^) were fixed. Appropriate amounts of BODIPY **1** photocatalyst were added to solutions of polymers. Mixing the solutions using a magnetic stirrer lasted 2 h until homogeneous solutions were obtained. Then electrospinning was performed. Details are provided in Table 1.

**Table 1:** Summary of the electrospinning process.

Sample ID	BODIPY addition [wt %]	Polymer concentration [%]	Solvents	Voltage (kV)	Tip-to-collector distance [mm]	Feed rate [mL·h^−1^]

PCL	—	12	AA, FA	12	100	1
**1**(0.15 wt %)@PCL	0.15	12	AA, FA	12	100	1
**1**(0.50 wt %)@PCL	0.50	12	AA, FA	12	100	1
**1**(1.00 wt %)@PCL	1.00	12	AA, FA	12	100	1
**1**(0.15 wt %)@PMMA	0.15	30	DMF	10	120	1
**1**(0.15 wt %)@PS	0.15	15	DMF, THF	20	150	2

All nanofibrous samples were deposited on aluminum foil (thickness of 30 µm, Rotilabo®, Roth Selection, Germany), which was attached to the collector. After the electrospinning process, all mats were placed under a fume hood for 48 h to evaporate solvents in the fibers.

### Morphology analysis

The morphological properties of samples were examined through scanning electron microscopy (SEM) using a JSM-6010PLUS/LV InTouchScope™ system from JEOL (Tokyo, Japan), operating at an accelerating voltage of 11 kV. Prior to imaging, each nonwoven sample underwent a double-coating process with a thin gold layer (2–3 nm). Microstructural analysis was subsequently performed utilizing ImageJ software (version 1.52q, USA). The fiber diameter distribution was evaluated based on a Gaussian approximation, with 100 measurements collected for each sample. The porosity (*p*) was calculated using Equation 1:


[1]

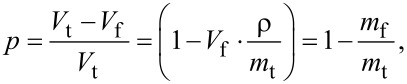



where *V*_f_ represents the volume of the tested sample, *m*_f_ is the sample’s mass, and *m*_t_ is the theoretical mass of the solid sample, calculated as the product of the density of PCL, 1.081 g·cm^−3^ [51], and the volume *V*_t_ ≈ 3.98 cm^3^. The pore size (*P*) was estimated using Equation 2:


[2]

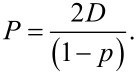



Here, *D* denotes the mean fiber diameter for each specimen, (1 − *p*) corresponds to the area of fibers per unit, and *p* indicates the porosity.

### Photocatalyst distribution in the mats

The presence of BODIPY in the PCL, PMMA, and PS matrices was verified using energy-dispersive X-ray spectroscopy (EDS) as well as time-of-flight secondary ion mass spectrometry (ToF SIMS) measurements performed on a Helios 5 Dual Beam microscope (ThermoFisher) equipped with a focused electron beam and a Xe ion beam. The EDS experiments were performed with a working distance of 4 mm, an accelerating voltage of 15 kV, a probe current of 0.2 nA, a magnification of 15000×, and a collection time of 30 min. Prior to ToF SIMS measurements, the samples were sputtered with 10 nm of gold using a CCU-010 Compact Coating Unit (Safematic). The ToF SIMS maps were acquired in the positive ion mode spectrum using 8 kV accelerating voltage and 10 nA beam current.

### X-ray photoelectron spectroscopy

X-ray photoelectron spectroscopy (XPS) analysis was done with an AXIS Supra+ (Kratos Analytical) instrument equipped with a monochromatic Al Kα X-ray source (h*ν* = 1486.6 eV, operating at 10 mA, 15 kV). The system base pressure was *p*_b_ = 3.9·10^−9^ Torr. The pass energy was set to 160 eV (scanning step 0.9 eV) for survey spectra acquisition and 20 eV (scanning step 0.05 eV) for high-resolution spectra acquisition. To compensate charging effects, the Kratos charge neutralization system was used. The binding energy scale was calibrated with respect to the C–C component of C 1s spectra (284.8 eV). The acquired spectra were analyzed using CASA XPS® software and embedded algorithms. The components of the high-resolution spectra were represented with Gaussian (70%) and Lorentzian (30%) lines, while the background was represented with Shirley’s function.

### Water contact angle measurements and surface free energy determination

The wettability of all samples was assessed using a Data Physics OCA 15EC contact angle goniometer (Filderstadt, Germany). A 2 µL droplet of liquid was placed on the scaffold surface, and the contact angle (CA) was measured after 3 s at a temperature of 21 °C.

The Owens–Wendt method was used to calculate the components of the surface free energy (SFE), which in its basic form employs two probe liquids with known polar and dispersive components. In this study, water and diiodomethane were used to determine the values of 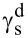 and 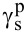, while formamide was applied as an additional reference liquid to verify the repeatability and reliability of the contact angle measurements (parameters related to formamide were not directly included in Equation 3 and Equation 4). The total surface free energy was calculated as the sum of the dispersive and polar components, 
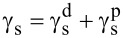
, where γ_s_ denotes the total surface free energy, and 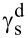 and 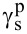 represent its dispersive and polar components, respectively [52–53] (Equation 3 and Equation 4):


[3]

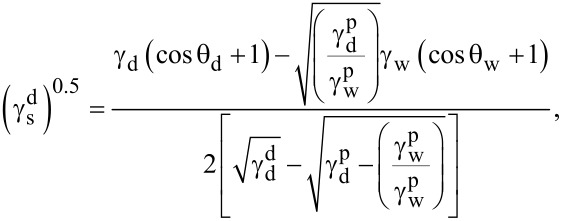




[4]

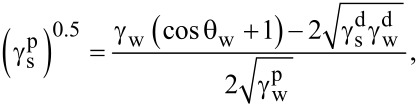



where 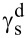 is the dispersion component of the SFE, 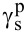 is the polar component of the SFE, γ_w_ is the SFE of water, 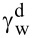 is its dispersive component, and 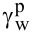 is its polar component; θ_w_ and θ_d_ are the CAs of water and diiodomethane, respectively. The surface polarity (*X*_p_) was determined using Wu’s method (Equation 5), with γ_s_ being the total surface free energy:


[5]

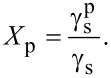



### Optical properties

The absorbance of the polymeric nonwovens doped with **1** was measured with an Evolution One Plus UV–vis spectrophotometer (Thermo Fisher Scientific) equipped with ISA-220 Integrating Sphere (reflectance measurements). Emission and fluorescence quantum yields of BODIPY fibers were determined using an Edinburgh Instruments FS5 spectrofluorometer. The measurements were performed at room temperature using solid-state Suprasil quartz cuvettes with front-face collection mode. The fluorescence quantum yield was measured with the integrating sphere according to the known procedures [54]. Fluorescence lifetime measurements were carried out using a time-correlated single-photon counting system equipped with a picosecond pulsed 340 nm EPLED source. The singlet oxygen emission was measured using a thermoelectrically cooled NIR-PMP unit (Hamamatsy, wavelength range 950–1650 nm) integrated in the FS5 spectrofluorometer.

### Photocatalytic degradation of API

Vials containing 1.5 mL of aqueous solutions of APIs (*c* = 1.05 mg·mL^−1^), tetrabutylammonium bromide (TBAB, *c* = 0.64 mg·mL^−1^, used as internal reference for ^1^H NMR analysis), and fragments of the photoactive material (5 × 5 mm) were placed in the photoreactor equipped with 26 W neutral-white light (CIE 1931 (0.38899; 0.37837)) LED stripes (Figure S5, Supporting Information File 1). The distance from the light source was the same for all samples (25 mm), providing the same irradiance of 40 mW·cm^−2^. The reactor was placed on a magnetic stirrer. Each vial was equipped with cross-shaped stirrer bars to ensure vigorous stirring. All experiments were repeated four times, and the results were calculated by mean ± standard deviation. Reaction progress was monitored by ^1^H NMR spectra analysis of the reaction mixture sampled after a given time. From each of the taken samples, the water was evaporated, D_2_O was added, and the composition of the mixture was investigated with ^1^H NMR.

### Photoinactivation studies

The photoinactivation potential of the fibers was evaluated on the Gram-positive bacterium *Staphyloccocus aureus* (ATCC 6538). For this assay, we used **1**(0.15 wt %)@PCL, **1**(0.50 wt %)@PCL, **1**(1.00 wt %)@PCL, and pure PCL for the reference. Bacteria were grown in Mueller–Hinton broth (BioMaxima, Poland). Inactivation studies were performed using two sterile, flat-bottom 24-well plates; one was illuminated and the second was used as the dark control. Materials were disinfected from wild bacteria and fungi by washing with 70% EtOH solution, followed by a twofold rinse with saline solution (0.9% NaCl). Three samples of each material were cut to fit the bottom of the well (ca. 1 cm). 200 μL aliquots of bacteria cell cultures in diluted saline solution (10000×) were transferred to the wells and incubated at 37 °C upon white light irradiation for 1 h. An analogous plate was placed for 1 h at 37 °C in an incubator shielded from light. Irradiation was performed with 26 W white-light LEDs glued to an aluminium plate (20 × 23 cm) mounted on threaded legs (Figure S6, Supporting Information File 1). In all experiments, the distance between the light source and the plate was fixed at 10 cm, ensuring homogeneous light distribution (40 mW·cm^−2^) without a measurable rise in temperature within the incubator. Afterwards, material circles were washed with 800 μL of saline, and the resulting bacterial solution was transferred to 96-well plates for further serial dilution, followed by plating on Mueller–Hinton agar medium (BioMaxima, Poland). After the incubation period (24 h at 37 °C), colony forming units (CFUs) were counted; the irradiated samples were compared to the dark control and a PCL material-free control (treated as 100% survival). Statistical significance of the results was assessed using variance analysis (ANOVA) supported by Tukey’s post hoc test.

## Results and Discussion

### Selection of the matrix material

**Figure 3:**
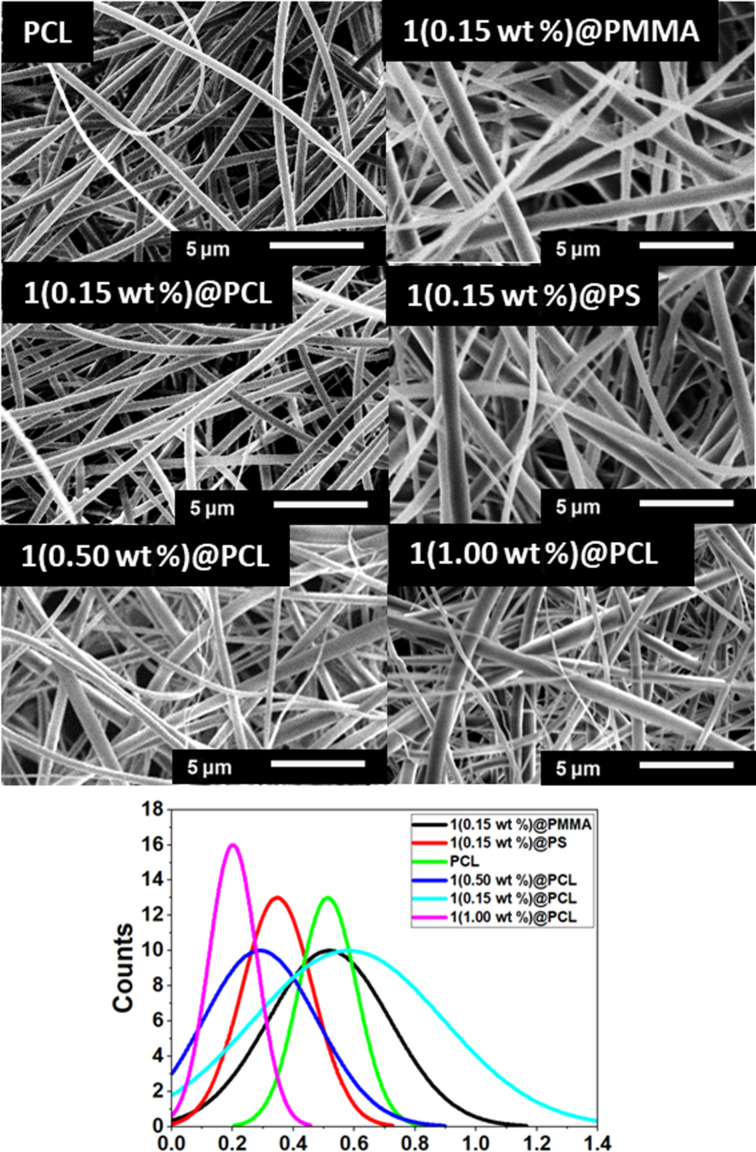
(Top) SEM images of electrospun mats with (bottom) diameter distributions approximated with Gaussian functions.

**Figure 4:**
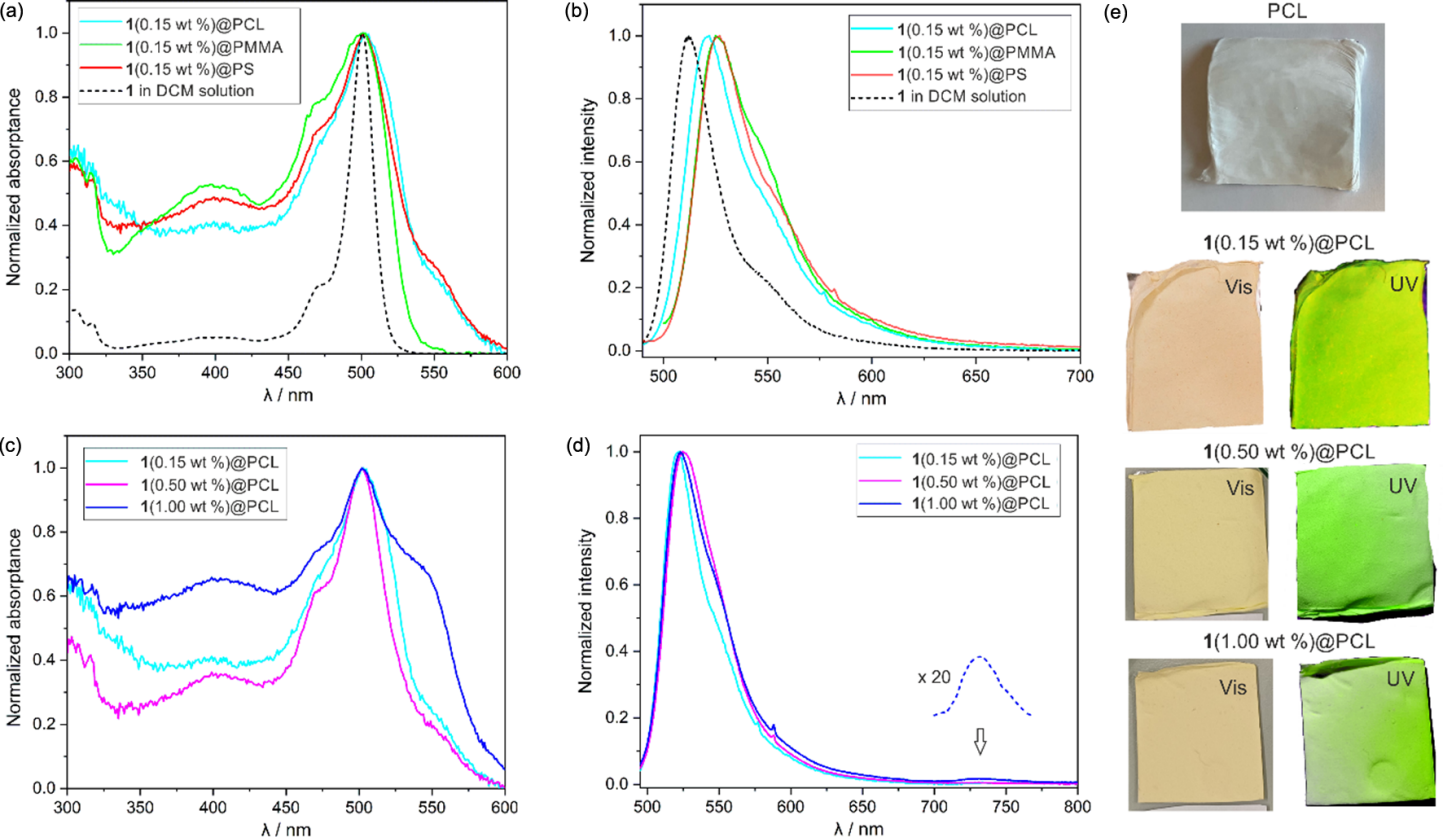
(a, c) Normalized absorption and (b, d) emission spectra (λ_ex_ = 470 nm) of the studied mats. (e) Photos of PCL and doped materials under visible and UV light.

Samples of BODIPY **1** encapsulated in poly(methyl methacrylate) (PMMA), polystyrene (PS), and polycaprolactone (PCL) nanofibers were formed using electrospinning, following by SEM analysis of the morphology. To ensure good homogeneity, the initial amount of the photocatalyst **1** was set to 0.15 wt %. The SEM microstructures revealed fibers with uniform and smooth morphology (Figure 3). The comparison among **1**(0.15 wt %)@PMMA, **1**(0.15 wt %)@PS, and **1**(0.15 wt %)@PCL revealed some differences in the average fiber diameter, decreasing from 600 ± 70 nm for **1**(0.15 wt %)@PCL to 390 nm for **1**(0.15 wt %)@PS. In addition, **1**(0.15 wt %)@PCL and **1**(0.15 wt %)@PMMA showed wider diameter distributions than **1**(0.15 wt %)@PS. The BODIPY-based mats absorb light at 450–550 nm, indicating that white light is a suitable excitation source. Overall, the absorbance spectra of all materials are relatively similar and closely align with the absorption maximum observed for the dilute solution in dichloromethane (DCM), but they display the band broadening characteristic of solid-state samples (Figure 4a). The emission spectra of the mats are slightly bathochromically shifted with respect to the DCM solution (Figure 4b). Specifically, the emission maximum of **1**(0.15 wt %)@PCL is centered at 521 nm (redshifted from 511 nm in DCM), while those of **1**(0.15 wt %)@PS and **1**(0.15 wt %)@PMMA are further shifted to 525 and 526 nm, respectively (Table 2).

**Table 2:** Emission properties of the studied materials.

Material	λ_abs_^a^	λ_em_^b^	QY^F c^

**1**(0.15 wt %)@PMMA	501	526	43%
**1**(0.15 wt %)@PS	502	525	35%
**1**(0.15 wt %)@PCL	501	521	8%
**1**(0.50 wt %)@PCL	501	525	26%
**1**(1.00 wt %)@PCL	501	523	21%
**1** (DCM solution) as reference	501	511	20%

^a^Absorbance wavelength maximum; ^b^emission wavelength maximum (λ_ex_ = 470 nm); ^c^total fluorescence quantum yield value.

In a subsequent step, we tested the photocatalytic activity of the prepared samples with cimetidine as a model water pollutant. Small pieces of materials were placed in 4 mL vials containing a 3.0 mM water solution of cimetidine. The mass of the material was ca. 0.011 g, which roughly corresponds to 0.02 equivalents of **1** photocatalyst per one equivalent of cimetidine. The reaction mixtures were vigorously stirred and irradiated with neutral-white LED light (26 W) at *T* = 25 °C in a photoreactor (irradiance of 40 mW·cm^−2^). The progress of the photocatalytic reaction was monitored using ^1^H NMR spectroscopy. The ^1^H NMR spectrum of cimetidine contains several signals that decrease as the reaction progresses, yielding numerous signals from unidentified decomposition products (Figure S2, Supporting Information File 1) [55]. Thus, to quantify the conversion with ^1^H NMR, all photocatalytic reactions were performed in the presence of tetrabutylammonium bromide (TBAB) as internal reference. TBAB does not absorb visible light, remains inactive in the presence of ROS, is not volatile, and gives a clear ^1^H NMR spectrum.

The photocatalytic experiments showed that cimetidine was quantitatively decomposed after 1 h with **1**(0.15 wt %)@PCL. The other two mats exhibited significantly lower activity, with the conversion of cimetidine reaching 20% for **1**(0.15 wt %)@PMMA and only 10% for **1**(0.15 wt %)@PS after 1 h. This can be attributed to their high hydrophobicity, as the poor wettability of these materials limits interphase contact. Interestingly, the fluorescence quantum yields of the PMMA- and PS-based materials reach 43% and 35%, respectively, substantially higher than that of **1**(0.15 wt %)@PCL (8%) (Table 2). This indicates that BODIPY **1** in the former matrices is more prone to deexcitation via prompt emission from the singlet state rather than to undergo intersystem crossing to the triplet state. Based on the initial assessment, PCL was selected as the optimal matrix for subsequent studies. Beneficially, PCL is easily accessible, non-toxic, and biodegradable, approved by the Food and Drug Administration (FDA) and European Medicines Agency (EMA); it is also commonly applied in medicine.

### Doping concentration effect in PCL-based materials

In the next step, we examined three different concentrations of **1** in the PCL matrixes, that is, 0.15, 0.50, and 1.00 wt %. The morphology of the mats was investigated with SEM, while fluorescence spectroscopy was used to check the possible aggregation of photosensitizer in PCL, as the emission behavior of BODIPY is sensitive to self-aggregation effects. The surface free energy value (SFE) was determined using the Kaelble–Owens–Wendt method, which accounts for both the polar component resulting from hydrogen and dipole–dipole interactions, as well as the dispersive component associated with London and van der Waals forces [56]. The study was complemented with X-ray photoelectron spectroscopy (XPS) for the sample with the highest BODIPY concentration, **1**(1.00 wt %)@PCL.

The analysis of SEM images showed that the increase in dye content led to a gradual decrease in the average PCL fiber diameter, from 600 ± 70 nm for **1**(0.15 wt %)@PCL to 210 ± 44 nm for **1**(1.00 wt %)@PCL (Figure 3). This effect may be due to changes in solution properties, such as a possible decrease in viscosity due to interactions between BODIPY molecules and PCL chains. Increasing the dye content promotes stronger jet stretching in the electric field during electrospinning, leading to the formation of thinner fibers. Porosity and average pore size analysis confirmed the effect of BODIPY addition, leading to increased porosity and slightly smaller pore sizes. For pure PCL, the porosity was 88.4%, while for PCL with BODIPY additive, the porosity increased to approximately 94%. The average pore size decreased from 204 ± 7 nm for pure PCL to 192 ± 4 nm after BODIPY addition. The observed increase of porosity is within the typical range for electrospun materials.

**Figure 5:**
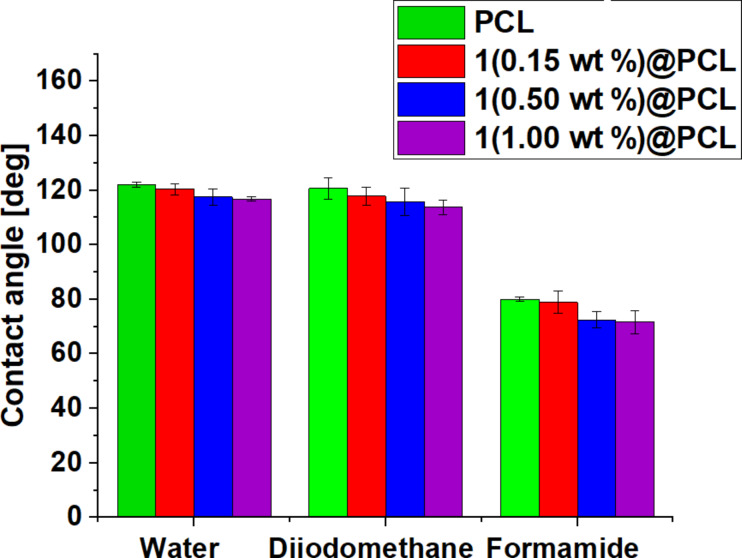
CA measurements of PCL mats.

The addition of highly hydrophobic BODIPY to the polymer fibers slightly reduced the hydrophilicity of the material. This is evidenced by a small but systematic decrease in the water contact angle (WCA), from 122° ± 0.9° for pure PCL to 117.49° ± 3.1° for **1**(0.50 wt %)@PCL and further to 116.79° for **1**(1.00 wt %)@PCL (Figure 5). The concentration of BODIPY also affects the surface free energy value (Table 3). It was observed that the dispersive component was the dominant factor influencing the SFE values of the tested substrates. The SFE of pure PCL was 27 ± 1.5 mN·m^−1^, while the SFE for the sample with the highest BODIPY addition (**1**(1.00 wt %)@PCL), was 44.08 ± 1.1 mN·m^−1^. In general, the SFE increased in the following order: PCL < **1**(0.15 wt %)@PCL < **1**(0.50 wt %)@PCL < **1**(1.00 wt %)@PCL. Among the tested materials, pure PCL exhibited the lowest polar component. Furthermore, the polar components increased with the addition of dyes, which was most effective in the case of **1**(1.00 wt %)@PCL. The addition of BODIPY increased the surface polarity of the samples due to the low polar component 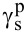 of approximately 3.9–6.7 mN·m^−1^ and the associated low WCA [57]. The enhancement of surface polarity with BODIPY incorporation is consistent with the observed decrease in WCA and may promote pollutant adsorption [58].

**Table 3:** Summary of water contact angle measurements and SFE analysis.

Sample	Water contact angle [°]	Dispersive component [mN·m^−1^]	Polar component [mN·m^−1^]	Surface free energy [mN·m^−1^]	Surface polarity [*X*_p_]

PCL	122 ± 0.9	23.3 ± 1.9	2.7 ± 0.6	28 ± 1.5	0.0964
**1**(0.15 wt %)@PCL	120.4 ± 2.1	29.8 ± 2.4	3.9 ± 0.9	33.7 ± 0.7	0.1157
**1**(0.50 wt %)@PCL	117.49 ± 3.3	30.2 ± 0.6	4.2 ± 0.3	34.4 ± 0.4	0.1220
**1**(1.00 wt %)@PCL	116.79 ± 0.79	37.38 ± 0.9	6.7 ± 0.8	44.08 ± 1.1	0.1519

**Figure 6:**
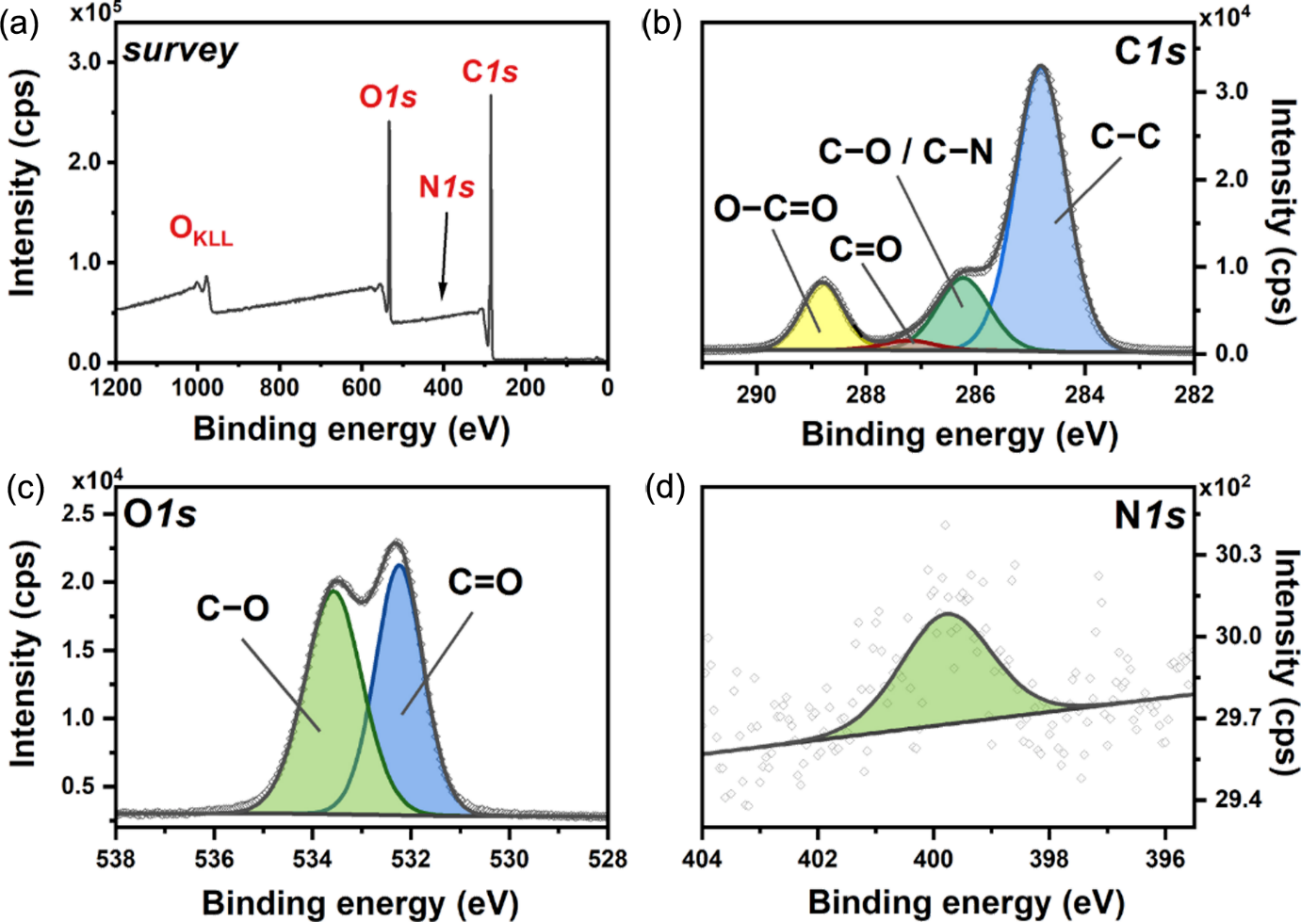
(a) Survey XPS spectrum and high-resolution spectra of (b) C 1s, (c) O 1s, and (d) N 1s energy regions recorded for **1**(1.00 wt %)@PCL.

**Figure 7:**
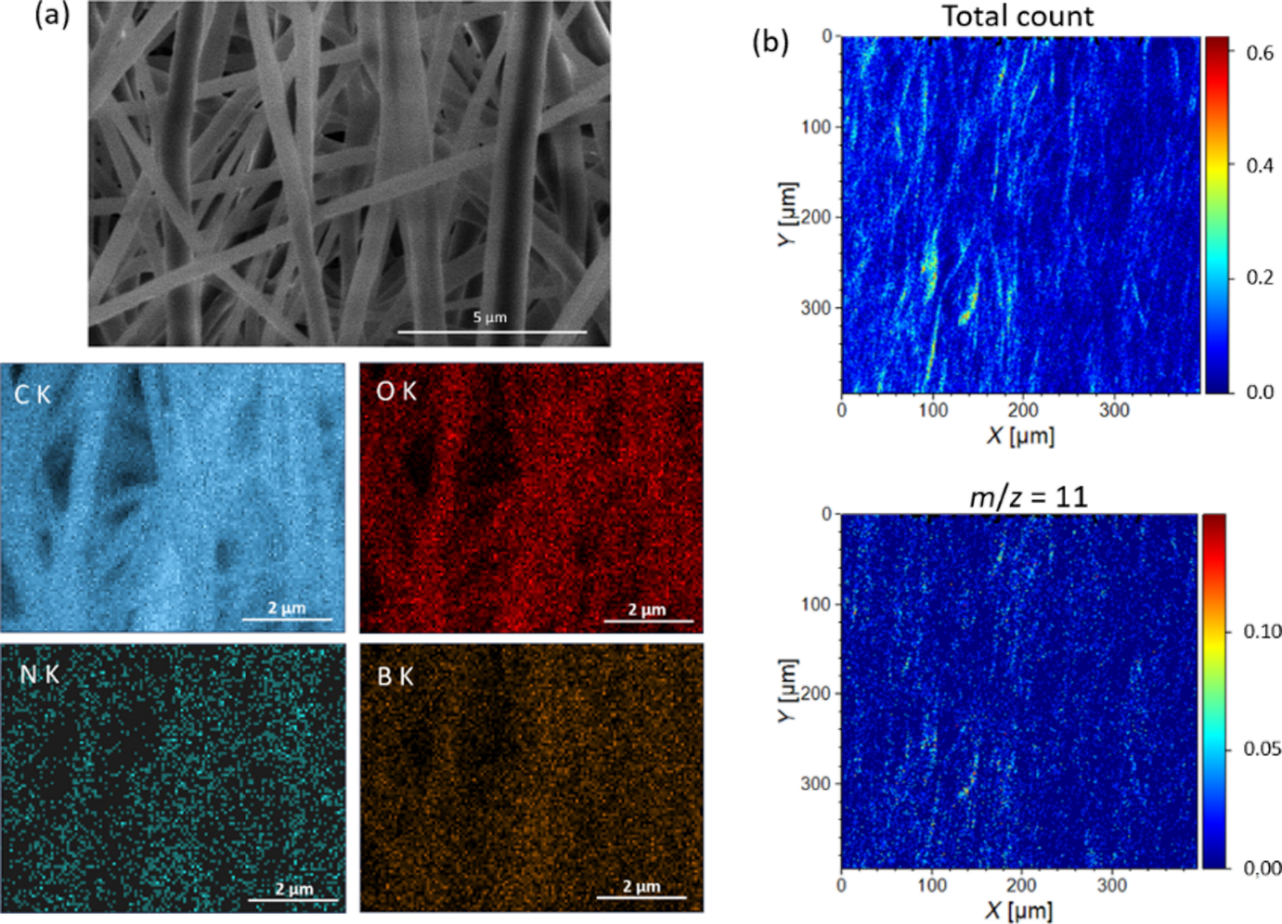
Images obtained for **1**(1.00 wt %)@PCL in (a) EDX measurements (SEM image and elemental maps of C, O, N, and B) and (b) ToF SIMS measurements (total ion count and *m*/*z* = 11 ion map).

The survey XPS spectrum recorded for **1**(1.00 wt %)@PCL (Figure 6a) confirms the presence of oxygen (O 1s at 530 eV, O_KLL_ at 970 eV), carbon (C 1s at 285 eV), and nitrogen (N 1s at 400 eV), while boron was not detected due to lower sensitivity of XPS for boron. The decomposition of the C 1s high-resolution spectrum (Figure 6b) gives four components located at 284.8, 286.2, 287.2, and 288.8 eV, which can be assigned to C−C, C−O/C−N, C=O, and O−C=O, respectively. While all components can be associated with PCL [59] modified with BODIPY [60], adventitious carbon contamination may also contribute to C−C, C−O, and C=O signals. The analysis of the O 1s high-resolution spectrum (Figure 6c) reveals the presence of two components, that is, C−O at 533.5 eV and C=O at 532.2 eV [59]. Finally, the N 1s high-resolution spectrum (Figure 6d) exhibits a single component with a maximum at 399.8 eV, confirming the presence of BODIPY [60]. The subsequent EDX analysis verified the presence of C, O, N, and B on the surface of the material and confirmed the uniform distribution of the BODIPY photocatalyst in PCL (Figure 7a). The distribution of boron in the sample was further verified using secondary ion mass spectrometry. The ion map of the main boron isotope with *m*/*z* = 11 is depicted in Figure 7b along with the total ion count map, confirming the isotropic distribution of **1** in the mats.

The absorbance spectra of the **1**(0.15 wt %)@PCL and **1**(0.50 wt %)@PCL are generally similar; however, for **1**(1.00 wt %)@PCL, the absorption band is significantly broadened, accompanied by a pronounced intensity increase in the right-band shoulder (Figure 4c). This spectral change is also reflected in the material’s apparent color, shifting to orange-reddish when the concentration increases (Figure 4e). The fluorescence behavior of all PCL-based mats is generally very similar and resembles the emission of the dilute DCM solution with the emission maximum slightly shifted from λ_em_(DCM) = 511 nm [34,61] to 521–525 nm (Table 2, Figure 4d). Fluorescence lifetime measurements acquired with a time-correlated single-photon counting system indicate a monoexponential decay (Figures S8–S10, Supporting Information File 1). The fluorescence lifetime oscillates close to 3 ns and is also comparable to the value measured for the DCM solution (2 ns). For **1**(1.00 wt %)@PCL, along with the emission band at 523 nm, a new small and broad emission band at 730 nm, attributed to the aggregate state, appeared. The fluorescence quantum yield of the **1**(0.15 wt %)@PCL mat measured using the integrating sphere is lower (QY^F^ = 8%) with respect to the DCM solution (QY^F^ = 20%). For **1**(0.50 wt %)@PCL, QY^F^ increases to 26%, to drop to 21% for **1**(1.00 wt %)@PCL. Phosphorescence was not detected in the materials, in accordance with the non-emissive nature of the triplet state in BODIPY.

### Photocatalytic properties of BODIPY@PCL materials

**Figure 8:**
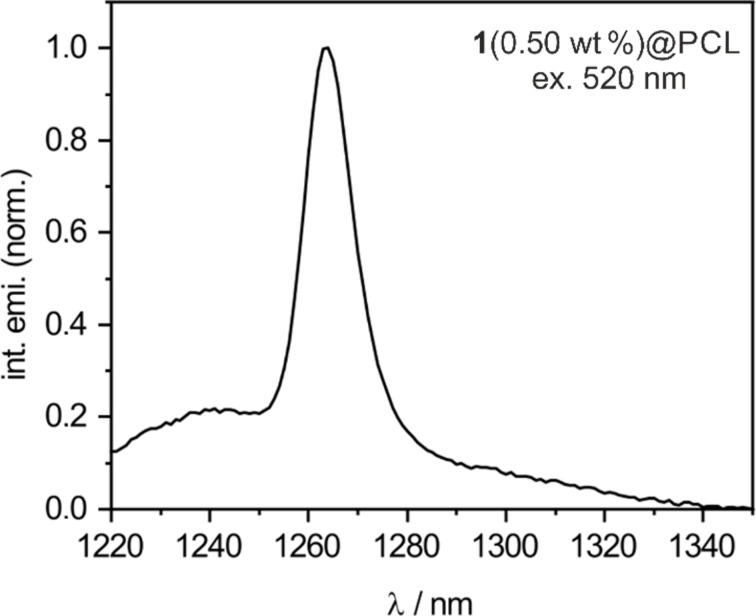
Emission spectrum of singlet oxygen for **1**(0.50 wt %)@PCL excited at λ_ex_ = 520 nm. The emission spectrum of singlet oxygen for PCL@**1**(1.00 wt %) is presented in Figure S7, Supporting Information File 1.

The generation of singlet oxygen in the studied mats was first confirmed by direct observation of the singlet oxygen phosphorescence (Figure 8). The materials were immersed in water in quartz cuvettes and irradiated at λ_ex_ = 520 nm, while recording the emission spectra in the NIR region. The immediate appearance of a distinct emission band at 1270 nm provides unequivocal evidence for the formation of singlet oxygen.

**Figure 9:**
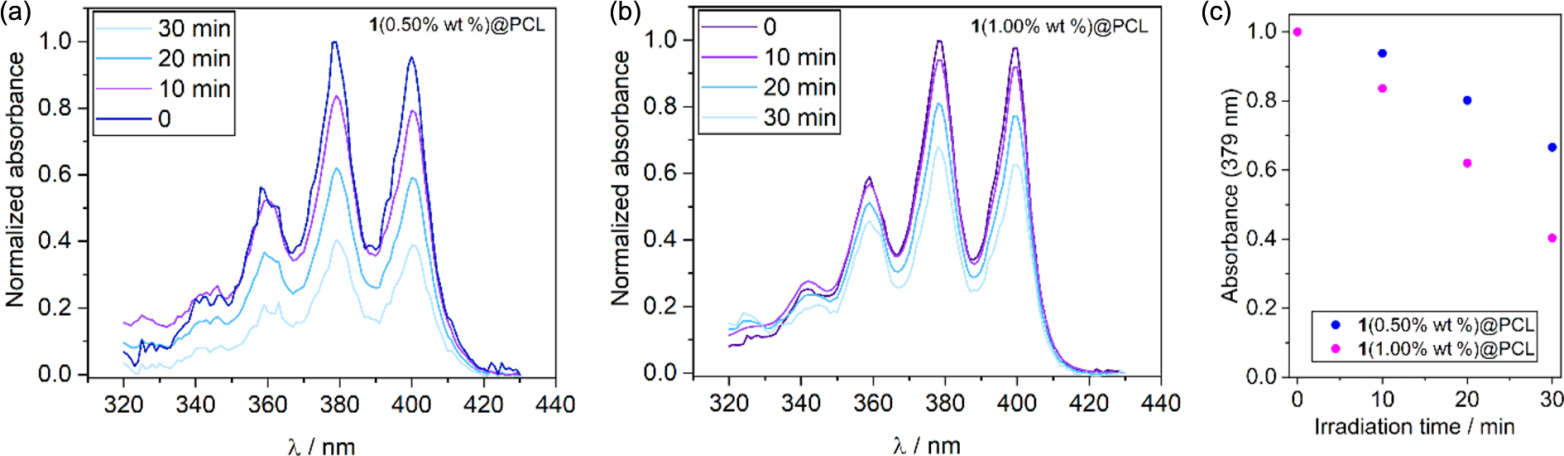
Changes in absorbance spectra of ABDA in PBS buffer solution in the presence of (a) **1**(0.50wt %)@PCL and (b) **1**(1.00wt %)@PCL irradiated with a white-light LED (40 mW·cm^−2^). (c) Drop in the absorbance at 379 nm after irradiation.

Next, we used phosphate-buffered saline (PBS) solution containing 10-anthracenediyl-bis(methylene) dimalonic acid (ABDA, *c* = 10^−4^ M) to trap singlet oxygen. The absorption spectra of the ABDA solution with immersed **1**(0.50 wt %)@PCL or **1**(1.00 wt %)@PCL were recorded after irradiation with white light (40 mW·cm^−2^) at 10 min intervals. After 30 min of irradiation, the absorbance at 379 nm decreased by 60% for **1**(0.50 wt %)@PCL and by 37% for **1**(1.00 wt %)@PCL (Figure 9). The higher activity of the former mat indicates triplet-state aggregation quenching at the higher dopant concentration.

The observed activity of the **1**@PCL mats was compared to electrospun PCL materials containing 2,6-dibrominated BF2-BODIPY at 0.05 wt % concentration studied by Cakmak and coworkers [62]. The authors demonstrated a drop of ABDA absorbance from 1.0 to ca. 0.7 after 30 min irradiation (530 nm LED, 100 mW·cm^−2^). In comparison, **1**(0.50 wt %)@PCL reduces the normalize absorbance of ABDA from 1.0 to 0.4 under white-light LED irradiation (40 mW·cm^−2^, 30 min). Considering the differences in concentrations and light irradiance, both materials show comparable photoactivity.

**Figure 10:**
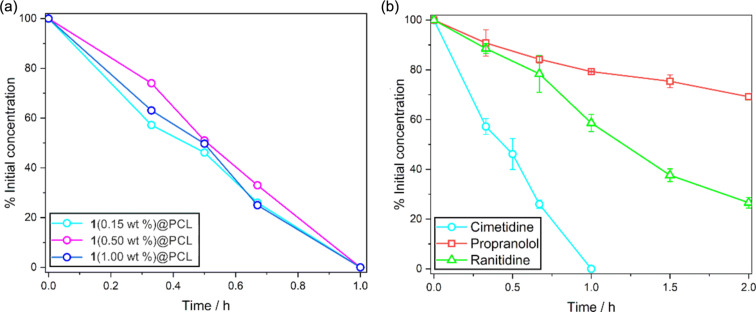
(a) Decomposition profiles of cimetidine with PCL-doped mats. (b) Decomposition profiles of model pharmaceutical pollutants with **1**(0.15 wt %)@PCL under white-light LED irradiation.

In the next step, the photocatalytic decomposition of cimetidine was studied at various doping concentrations. The reaction profiles are very similar (Figure 10a), indicating that the reaction rate is not limited by the concentration of **1**, but rather by the diffusion rate of oxygen. It should be also noted that **1**(0.50 wt %)@PCL exhibits higher fluorescence quantum yield (QY^F^ = 26%) than **1**(1.00 wt %)@PCL (QY^F^ = 21%), despite similar photocatalytic performance. This indicates that, at higher concentrations, the non-radiative processes may be more efficient due to the possible aggregation effects.

To further examine the performance of the electrospun photoactive fibers, we have conducted photocatalytic decontamination experiments involving two additional pharmaceutical agents, namely, ranitidine and propranolol. For these studies, we selected **1**(0.15 wt %)@PCL as it represents the most economical material option. The reaction profiles are depicted in Figure 10b. It turned out that the half-decomposition times of ranitidine and propranolol are 72 min and 4 h, respectively. We have also confirmed that the reaction does not proceed for pure PCL material nor in the absence of light, underscoring the key role of the BODIPY complex in the photogeneration of ROS.

The degradation of APIs with ROS is non-selective, involving multiple parallel pathways where intermediates may undergo further oxidation. Singlet oxygen is a strong electrophile engaging in [4 + 2] Diels–Alder cycloadditions with dienes to form endoperoxides, [2 + 2] cycloadditions with alkenes to yield dioxetanes, and ene reactions with allylic hydrogens to produce hydroperoxides; it also oxidizes sulfides to sulfoxides [63]. According to Quaresma and coworkers [64], cimetidine undergoes initial S-oxidation to the sulfoxide followed by C–S bond cleavage to yield (5-methyl-1*H*-imidazol-4-yl)methanol. A similar pathway is expected for ranitidine, which also incorporates a sulfur atom in the structure. Propranolol oxidation with ROS produces 4-hydroxypropranolol, α-naphthoxylactic acid, and other oxygenated products. Finally, the inspection of ^1^H NMR spectra upon photocatalytic reaction (Figures S2–S4, Supporting Information File 1) revealed that the total integral number of the decomposition products does not agree with the total number of reacted protons in the APIs, suggesting the paramagnetic nature of some of the decomposition products.

**Figure 11:**
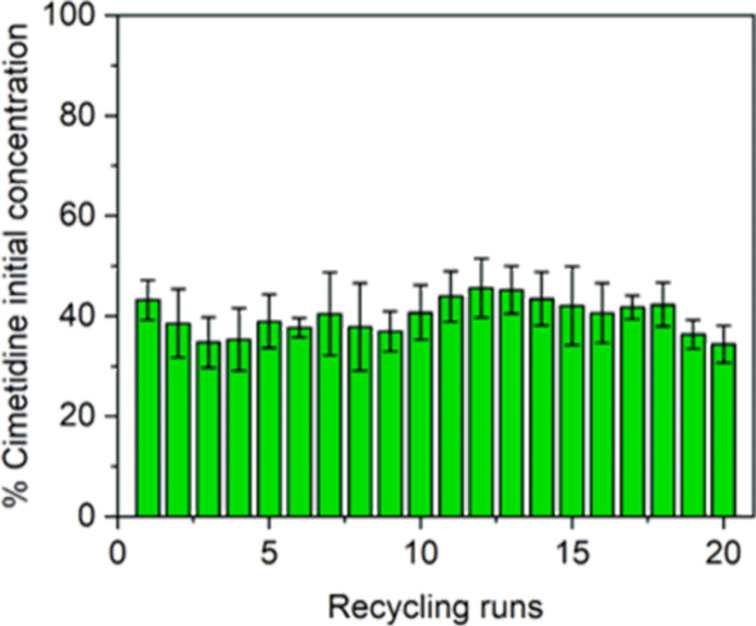
Photocatalytic cycles of **1**(0.15 wt %)@PCL with cimetidine as model contaminant. Single reaction time: 30 min.

The catalyst re-usability of **1**(0.15 wt %)@PCL with cimetidine was assessed by using the same material in 20 consecutive photocatalytic cycles. Each cycle lasted 0.5 h, which gives a total of 10 h of constant work of photocatalyst. After irradiation, the liquid phase was removed from the vial, the material was washed with distilled water, dried under reduced pressure (1 mbar, 25 °C, 1 h), and used in the next run without any additional treatment. Figure 11 shows some oscillations in the decomposition rates, but this is probably caused by the nature of the experiment, as the performance of the materials may vary to some extent due to the various positioning of the material in the vial. Nonetheless, these results demonstrate the high durability of the material, maintaining its activity even after 20 cycles.

We have also checked the possible leaching of the BODIPY photosensitizer from the nonwoven materials **1**(0.15 wt %)@PCL, **1**(0.50 wt %)@PCL, and **1**(1.00 wt %)@PCL. The materials (12 mg) were immersed in 1.5 mL of water and stirred for 24 h; then, the water was filtered, the fabric was washed with water, and the combined water phase was extracted with CHCl_3_ followed by UV–vis spectroscopy measurements. Each experiment was repeated four times. Our results show that the average daily BODIPY leaching is below 0.1%, confirming the good compatibility of **1** and PCL.

### Photoinactivation of microbes

**Figure 12:**
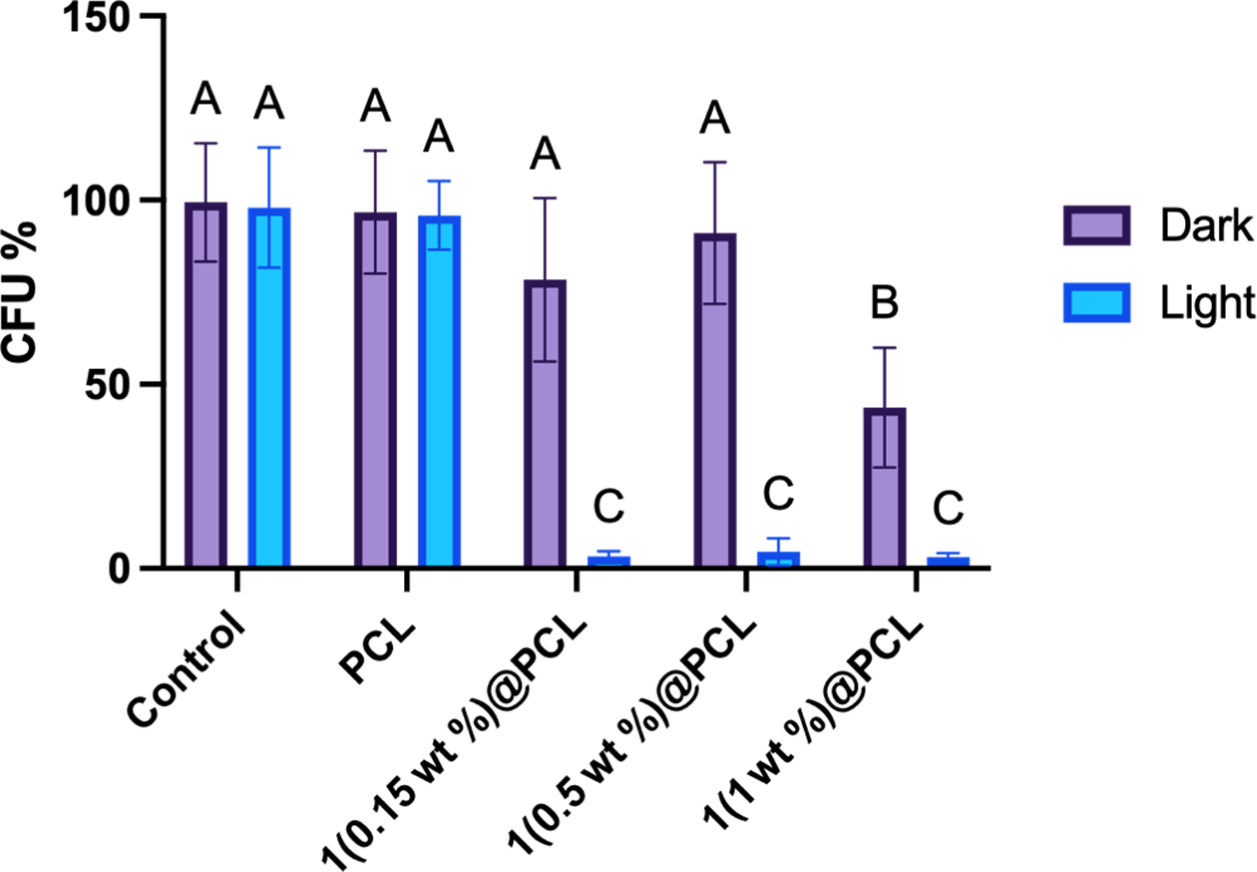
Comparison of CFUs of *Staphyloccocus aureus* growing on nonwovens with photosensitizer at different concentrations (0.15, 0.50, and 1.00 wt %) exposed to white light irradiation for 2 h or kept in dark. Results are presented as percentage of control CFUs. Significant effect: values that do not share a common letter differ significantly; all significant differences are *P* ≤ 0.001.

Finally, we investigated the photoactivity of the materials against pathogenic bacteria. A series of antimicrobial assays were performed for all tested PCL mats with *S. aureus* selected as a model pathogen. The results of the CFU assay are presented in Figure 12. The plain PCL materials, both kept in the dark and exposed to white light irradiation, did not affect the CFUs of *Staphyloccocus aureus*. Likewise, the **1**(0.15 wt %)@PCL and **1**(0.50 wt %)@PCL samples kept under dark conditions did not cause a decrease in *S. aureus* CFUs, while **1**(1.00 wt %)@PCL showed some cytotoxic effect without irradiation. Importantly, our results clearly show that every studied concentration significantly decreased *S. aureus* CFUs under light irradiation as compared to the experiments performed without light. No clear concentration-dependent effect on CFU reduction was observed. Therefore, using a lower concentration of **1** can provide an antimicrobial efficacy similar to that of higher concentrations while potentially reducing toxicity and improving cost efficiency. The photoactivity of **1**@PCL materials is comparable to that of a BODIPY-embedded electrospun PCL material studied by Cakmak et al. [62], which achieved 0.6–2.6 log reductions in viability of the Gram-negative bacterium *Pseudomonas aeruginosa* upon 2 h of irradiation with a green LED (530 nm, 40 mW·cm^−2^).

## Conclusion

Our study demonstrates the first utilization of a heavy-atom-free BODIPY photosensitizer (**1**) as a component of a photoactive nanofiber material. Polycaprolactone was selected as optimal matrix for the immobilization of **1** due to its higher hydrophilicity and wettability with respect to poly(methyl methacrylate) and polystyrene. Remarkably, the **1**@PCL materials enabled complete degradation of cimetidine, selected as a model pollutant from pharmaceutical industry wastewater, within 1 h of white-light LED irradiation, even at a low photocatalyst loading of 0.15 wt %. SEM, EDX, ToF SIMS, and XPS analyses confirmed that the electrospun nanofibers have smooth, uniform morphologies with homogeneous distributions of **1**. The material showed very high durability, exhibiting excellent oxidation resistance without losing catalytic activity over 20 consecutive cycles. All tested PCL-based materials show high antimicrobial activity. Among them, **1**(0.50 wt %)@PCL was chosen as the optimal formulation; it did not hinder the growth of *S. aureus* in the dark but triggered a substantial decrease (to less than 5%) in bacterial growth under light irradiation. Overall, our results demonstrate the significant promise of electrospun BODIPY-based nanofibers for water purification, providing a durable and efficient platform for advanced photocatalytic treatment of pharmaceutical contaminants and pathogens.

## Supporting Information

File 1 Additional experimental data.

## Data Availability

The data supporting this article have been included as part of the Supporting Information. Detailed experimental data are available upon request from the authors.
