# Supplementary material for: Decontamination from water pollutants and pathogens by electrospun nanofibers doped with heavy-atom-free borafluorene-BODIPY photosensitizers
Source: Beilstein J Nanotechnol. 2026 May 20;17:668–82. doi: 10.3762/bjnano.17.46 (PMC13202474; doi:10.3762/bjnano.17.46)
Supplement: File 1 — Additional experimental data. [file Beilstein_J_Nanotechnol-17-668-s001.pdf]

BEILSTEIN JOURNAL OF NANOTECHNOLOGY

Supporting Information

for

Decontamination from water pollutants and pathogens by electrospun nanofibers doped with heavy-atom-free borafluorene-BODIPY photosensitizers

Angelika Zaszczyńska, Paulina H. Marek-Urban, Karolina Wrochna, Agnieszka E. Kuklewska, Kacper Kręgielewski, Marta Grodzik, Dawid R. Natkowski, Jolanta Mierzejewska, Ewa Iwanek, Agata Blacha-Grzechnik, Paweł Sajkiewicz and Krzysztof Durka

Beilstein J. Nanotechnol. **2026,** *17,* 668–682. doi:10.3762/bjnano.17.46

Additional experimental data

License and Terms: This is a supporting information file under the terms of the Creative Commons Attribution License (https://creativecommons.org/licenses/by/4.0). Please note that the reuse, redistribution and reproduction in particular requires that the author(s) and source are credited and that individual graphics may be subject to special legal provisions.
The license is subject to the *Beilstein Journal of Nanotechnology* terms and conditions: (https://www.beilstein-journals.org/bjnano/terms)

1 NMR

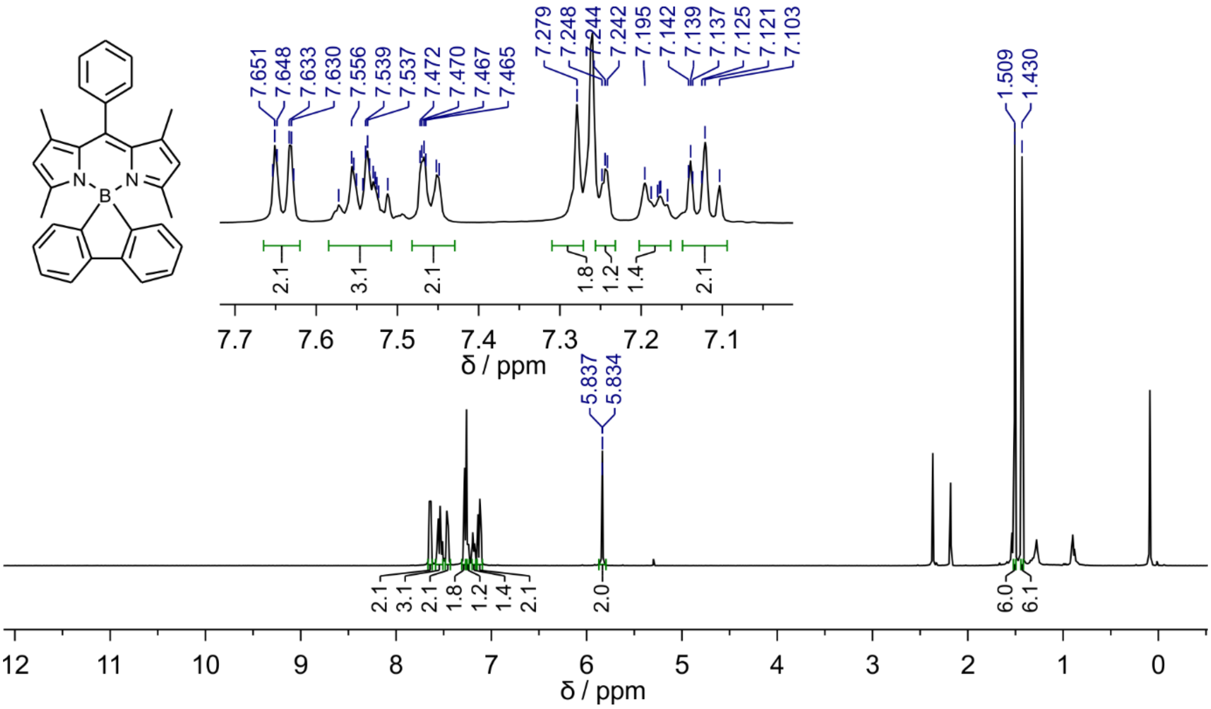

Figure S1: ^{1}H NMR (400 MHz) spectrum of **1** in $CDCl_3$.

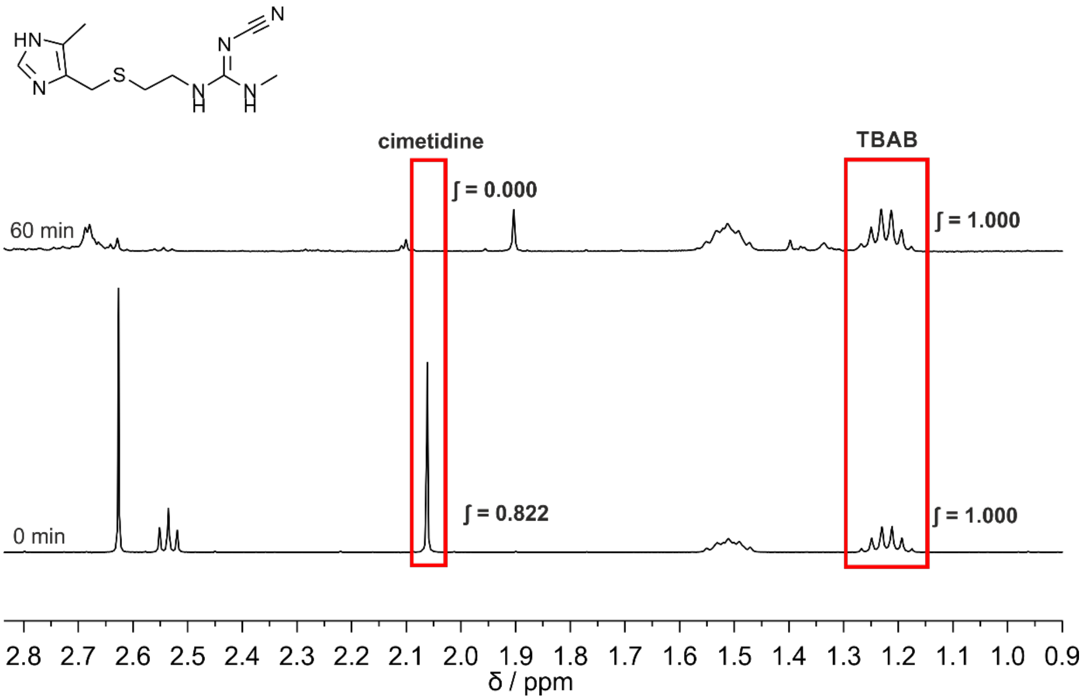

Figure S2: Analysis of the ^{1}H NMR spectrum (400 MHz, $CDCl_3$) for the photocatalytic oxidation of cimetidine with **1** (0.15 wt %)@PCL (H_2O, 26 W white LED, 25 °C). Integrals used for determination of reaction conversion are marked.

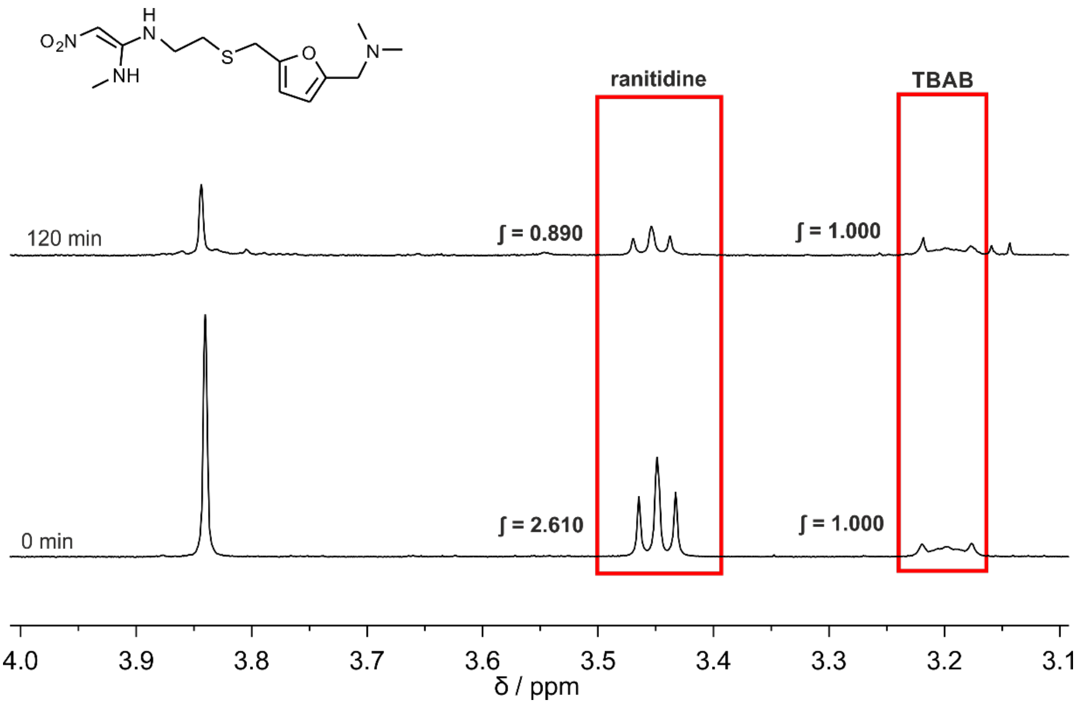

Figure S3: Analysis of the ^{1}H NMR spectrum (400 MHz, $CDCl_3$) for the photocatalytic oxidation of ranitidine with **1** (0.15 wt %)@PCL (H_2O, 26 W white LED, 25 °C). Integrals used for determination of reaction conversion are marked.

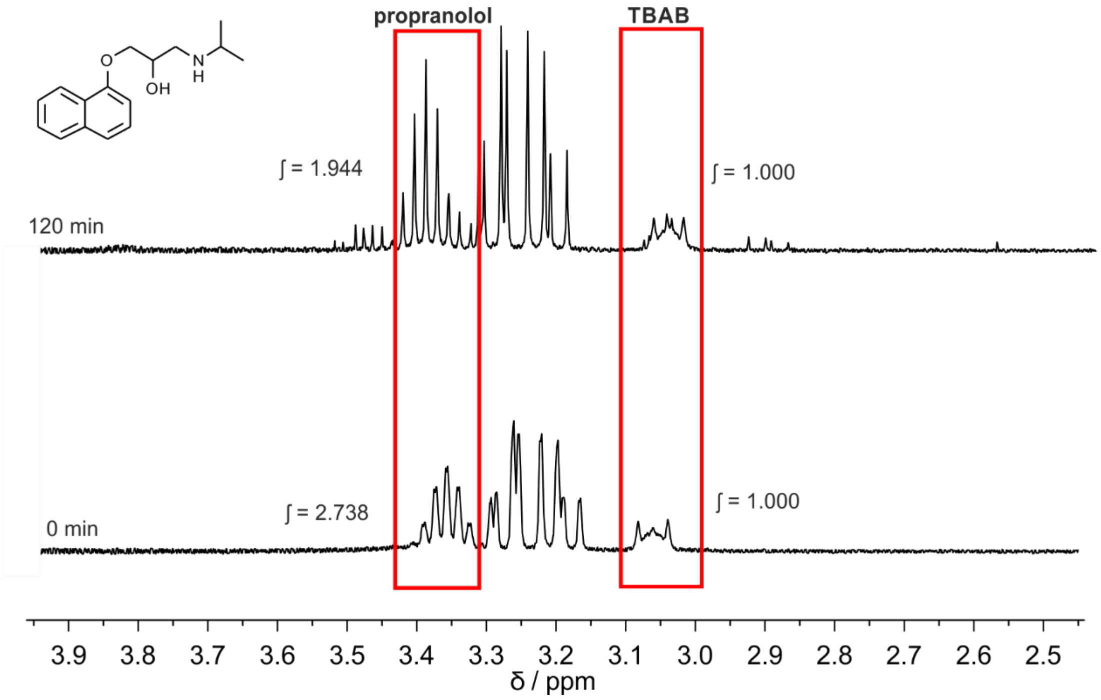

Figure S4: Analysis of the ^{1}H NMR spectrum (400 MHz, $CDCl_3$) for the photocatalytic oxidation of propranolol with **1**(0.15 wt %)@PCL (H_2O, 26 W white LED, 25 °C). Integrals used for determination of reaction conversion are marked.

2 Photocatalytic studies

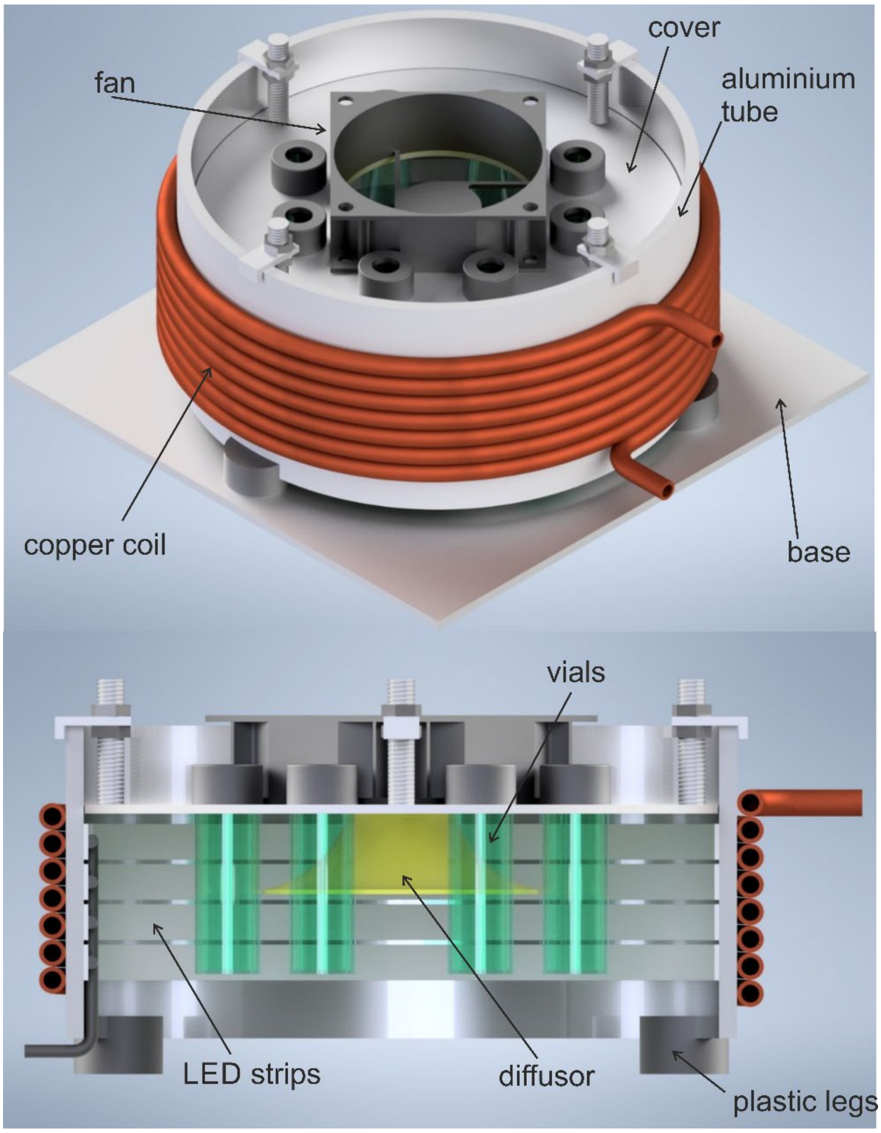

Figure S5: Model of photoreactor used in the photocatalytic degradation of pharmaceuticals. Figure S5 was adapted from [1], (© 2021 P. H. Marek-Urban et al., Published by American Chemical Society, distributed under the terms of the Creative Commons Attribution 4.0 International License, https://creativecommons.org/licenses/by/4.0).

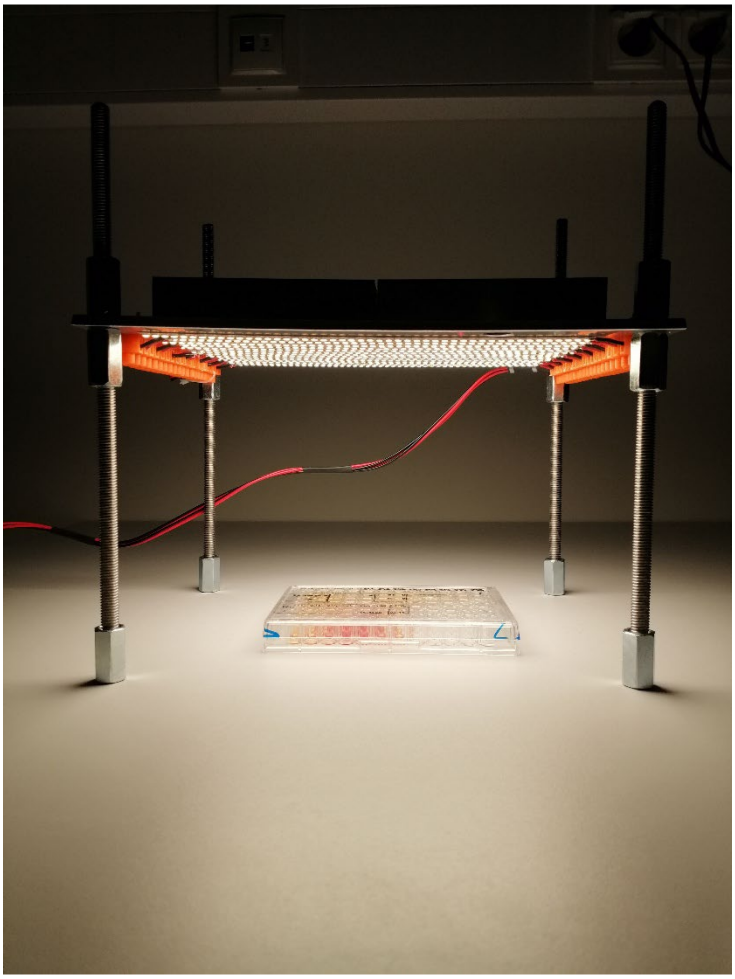

Figure S6: Photoreactor used in the microbiological experiments.

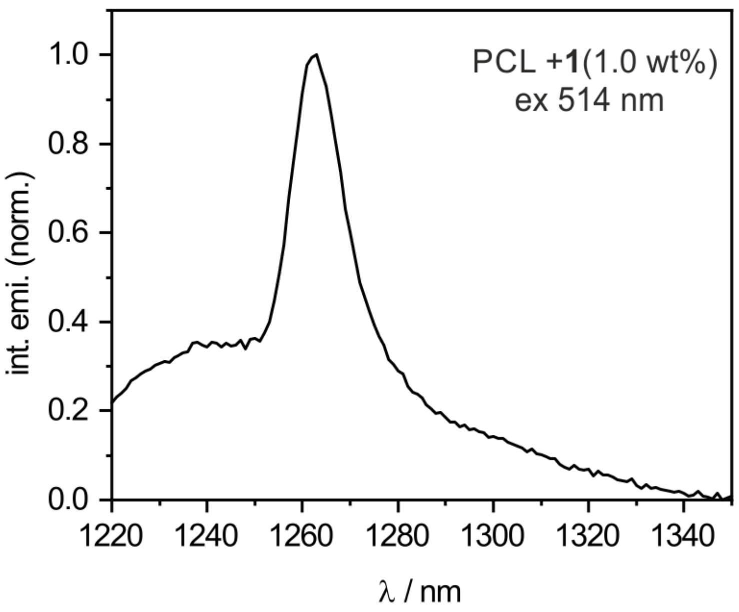

Figure S7: Emission spectrum of singlet oxygen for PCL@**1**(1.00 wt %) excited at λ_{ex} = 520 nm.

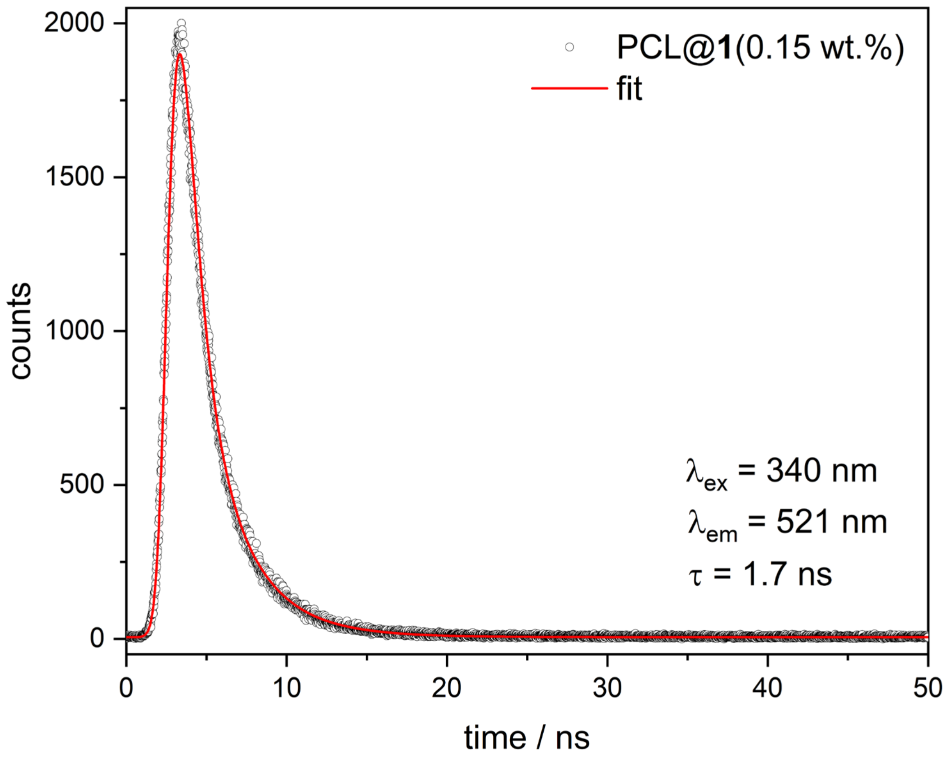

Figure S8: Fluorescence decay of PCL@**1**(0.15 wt %) at λ_{em} = 521 nm.

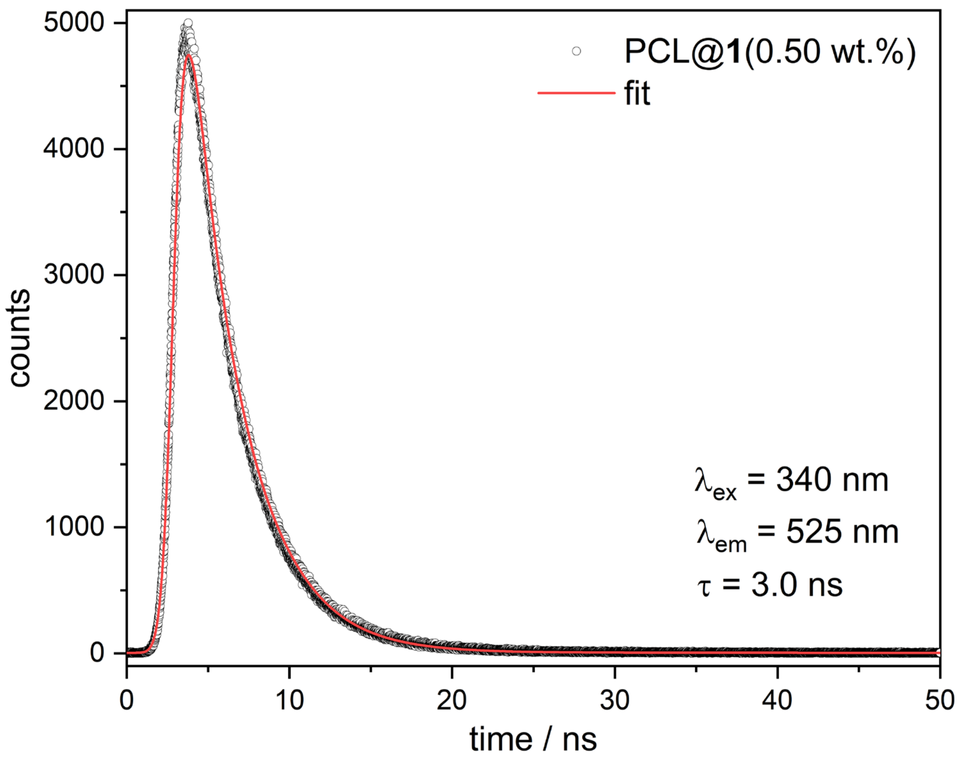

Figure S9: Fluorescence decay of PCL@**1**(0.50 wt %) at λ_{em} = 525 nm.

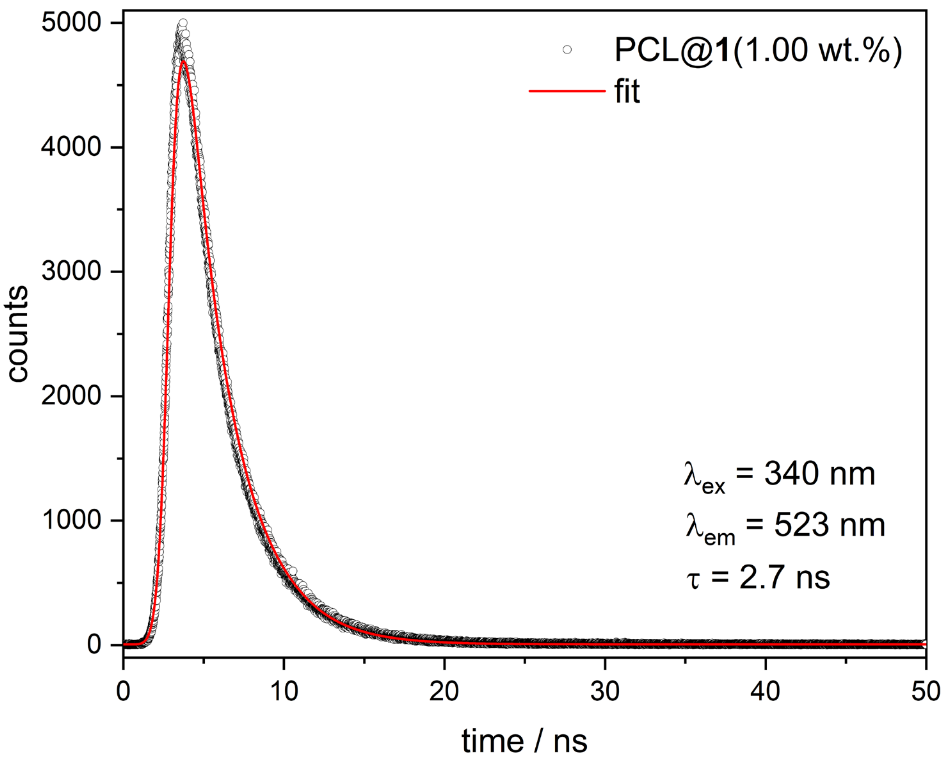

Figure S10: Fluorescence decay of PCL@**1**(1.00 wt %) at λ_{em} = 523 nm.

References

1. Marek-Urban, P.H.; Urban, M.; Wiklińska, M.; Paplińska, K.; Woźniak, K.; Blacha-Grzechnik, A.; Durka, K. *J. Org. Chem.* **2021,** *86,* 12714–12722. doi:10.1021/acs.joc.1c01254
